# A novel theory for rapid localization of the transverse-sigmoid sinus junction and “keyhole” in the retrosigmoid keyhole approach: micro-anatomical study, technique nuances, and clinical application

**DOI:** 10.1007/s10143-024-02583-x

**Published:** 2024-07-15

**Authors:** Zhi-Heng Jian, Min-Feng Sheng, Chang-Chun Liao, Zhi-Jian Weng, Jia-yan Li, Xin-Feng Yi, Gang Chen

**Affiliations:** 1 Neurosurgery Department, Zhuhai People’s hospital (Zhuhai Clinical Medical College of Jinan University, Zhuhai, Guangdong Province China; 2 Second affiliated hospital of Soochow University, Suzhou, Jiangsu Province China

**Keywords:** Retrosigmoid Approach, Micro-anatomy, Localization, Keypoint, Microsurgery

## Abstract

To determine a rapid and accurate method for locating the keypoint and “keyhole” in the suboccipital retrosigmoid keyhole approach. (1) Twelve adult skull specimens were selected to locate the anatomical landmarks on the external surface of the skull.The line between the infraorbital margin and superior margin of the external acoustic meatus was named the baseline. A coordinate system was established using the baseline and its perpendicular line through the top point of diagastric groove.The perpendicular distance (x), and the horizontal distance (y) between the central point of the “keyhole” and the top point of the digastric groove in that coordinate system were measured. The method was applied to fresh cadaveric specimens and 53 clinical cases to evaluate its application value. (1) x and y were 14.20 ± 2.63 mm and 6.54 ± 1.83 mm, respectively (left) and 14.95 ± 2.53 mm and 6.65 ± 1.61 mm, respectively (right). There was no significant difference between the left and right sides of the skull (*P* > 0.05). (2) The operative area was satisfactorily exposed in the fresh cadaveric specimens, and no venous sinus injury was observed. (3) In clinical practice, drilling did not cause injury to venous sinuses, the mean diameter of the bone windows was 2.0–2.5 cm, the mean craniotomy time was 26.01 ± 3.46 min, and the transverse and sigmoid sinuses of 47 patients were well-exposed. We propose a “one point, two lines, and two distances” for “keyhole” localization theory, that is we use the baseline between the infraorbital margin and superior margin of the external acoustic meatus and the perpendicular line to the baseline through the top point of the digastric groove to establish a coordinate system. And the drilling point was 14.0 mm above and 6.5 mm behind the top point of the digastric groove in the coordinate system.

**Supplementary Information:**

The online version contains supplementary material available at 10.1007/s10143-024-02583-x.

## Introduction

The suboccipital retrosigmoid approach is a classic surgical approach for treating cerebellopontine angle diseases [1–3]. Due to the natural barrier of the transverse and sigmoid sinuses, choosing the location of the bone window during craniotomy is very important. To achieve maximal exposure of the operative area, the bone window should be as close as possible to the inferior border of the transverse sinus and the posterior border of the sigmoid sinus [4]. With deepening of minimally invasive concepts, small incisions, micro-bone windows, and reduced bone flaps have been gradually recognized by neurosurgeons [5–7]. To reduce skin and bone damage, accurate identification of the keypoint, which is the extracranial point corresponding to the inferior margin of the transverse sinus–the posterior margin of the sigmoid sinus junction (TSJ), and the “keyhole,” which is the burr hole in craniotomy, is particularly important [8]. By analyzing the relationship between anatomical landmarks of skull specimens and verifying them with that of cadaveric specimens, previously we have reported a primary keyhole locating method [9], however in few clinical practice, the accuracy was not satisfactory. This study intended to develop a more precise method for locating the keypoint and “keyhole” (drilling point) to provide a quantitative basis for safe, accurate, and rapid craniotomy using the suboccipital retrosigmoid approach.

## Methods

### Specimens and instruments

Twelve adult skull specimens and five adult fresh cadaveric specimens from University were used in this study. No obvious deformities, trauma, or surgical traces were observed in the posterior cranial fossa or in the petrous and mastoid parts of the temporal and occipital bones. We used a microscope (OPMI PROergo, Carl Zeiss Meditec AG, Germany), dynamic craniotomy system (Microspeed Uni GD670; Aesculap, Germany), straight scale (DL8050, Deli Group Co., Ltd., China), digital display Vernier calipers (AS0020151, Shanghai tool factory, China), digital display angle scale (Casillo 0–200 mm, Henan Bangte Measuring Tools Co., Ltd., China), homemade head racks, and microsurgical instruments.

### Patient cohort

Fifty-three (22 men and 31 women aged 29 to 72 years (mean: 50.13 ± 10.17) patients from Hospital were involved in this research. Between June 2020 to June 2022, the patients underwent accurate craniotomy using the retrosigmoid approach conducted by a senior neurosurgeon. The studies involving human participants were reviewed and approved by the ethics committee of Hospital. The patients/participants provided written informed consent to participate in this study and they consented to the publication of their images. Preoperative image data of patients were obtained from the Department of Radiology in the hospital, and multimodal image fusion and 3D reconstruction of the cerebral, cerebellum, brainstem, cranial nerves, blood vessels, tumors and skull were performed using RadiAnt DICOM Viewer (Medixant, Poznan, Poland) or 3D slicer software(4.11.20210226). A total of 7, 19, 1, 1, and 25 patients were diagnosed with vestibular neuroma, trigeminal neuralgia, meningioma, schwannoma in the jugular foramen area, and hemifacial spasm, respectively.(Table 1).

**Table 1:** Characteristic of 53 clinical patients

Characteristic	Value
**Sex**	
Men	22
Women	31
Age (mean ± standard deviation)	50.13 ± 10.17
**Side**	
Right	22
Left	31
**Diagnosis**	
Acoustic neuroma	7
Trigeminal neuralgia	19
Meningioma	1
Hemifacial spasm	25
Jugular foramen schwannoma	1

### Experimental methods

#### Location of the keypoint and measurement of landmarks in the dry skulls

**Fig. 1:**
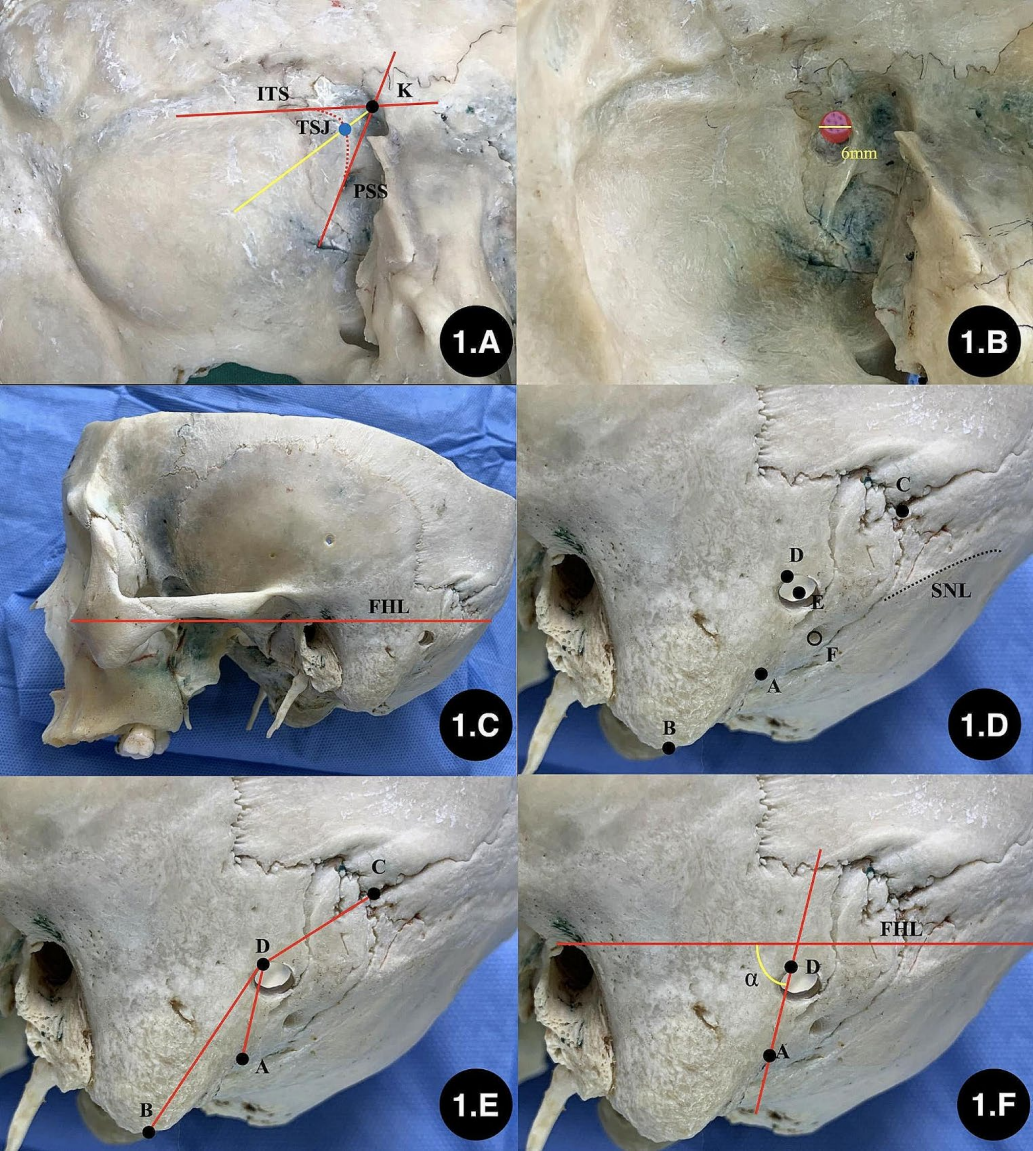
The method of locating the keypoint and “keyhole”. **A**: location of the TSJ; **B**: drilling the “keyhole” (6 mm in diameter); **C**: confirming the baseline; **D**: marking the extracranial anatomical landmarks; **E**: measuring the length between the anatomical landmarks; **F**: measuring the degree of ∠α. A: the top point of the digastric groove; B: mastoidale; C: asterion; D: keypoint; E: the central point of “keyhole”; F: mastoid foramen; K: intersection point of tangent line between inferior margin of transverse sinus and posterior margin of sigmoid sinus; TSJ: the inferior margin of the transverse sinus–medial margin of the sigmoid sinus junction; ITS: tangent line of inferior margin of transverse sinus; PSS: tangent line of posterior margin of sigmoid sinus; SNL: superior nuchal line; FHL: the baseline between the infraorbital margin and superior margin of the external acoustic meatus; ∠α: the anterior inferior angle between AD and the baseline

**Fig. 2:**
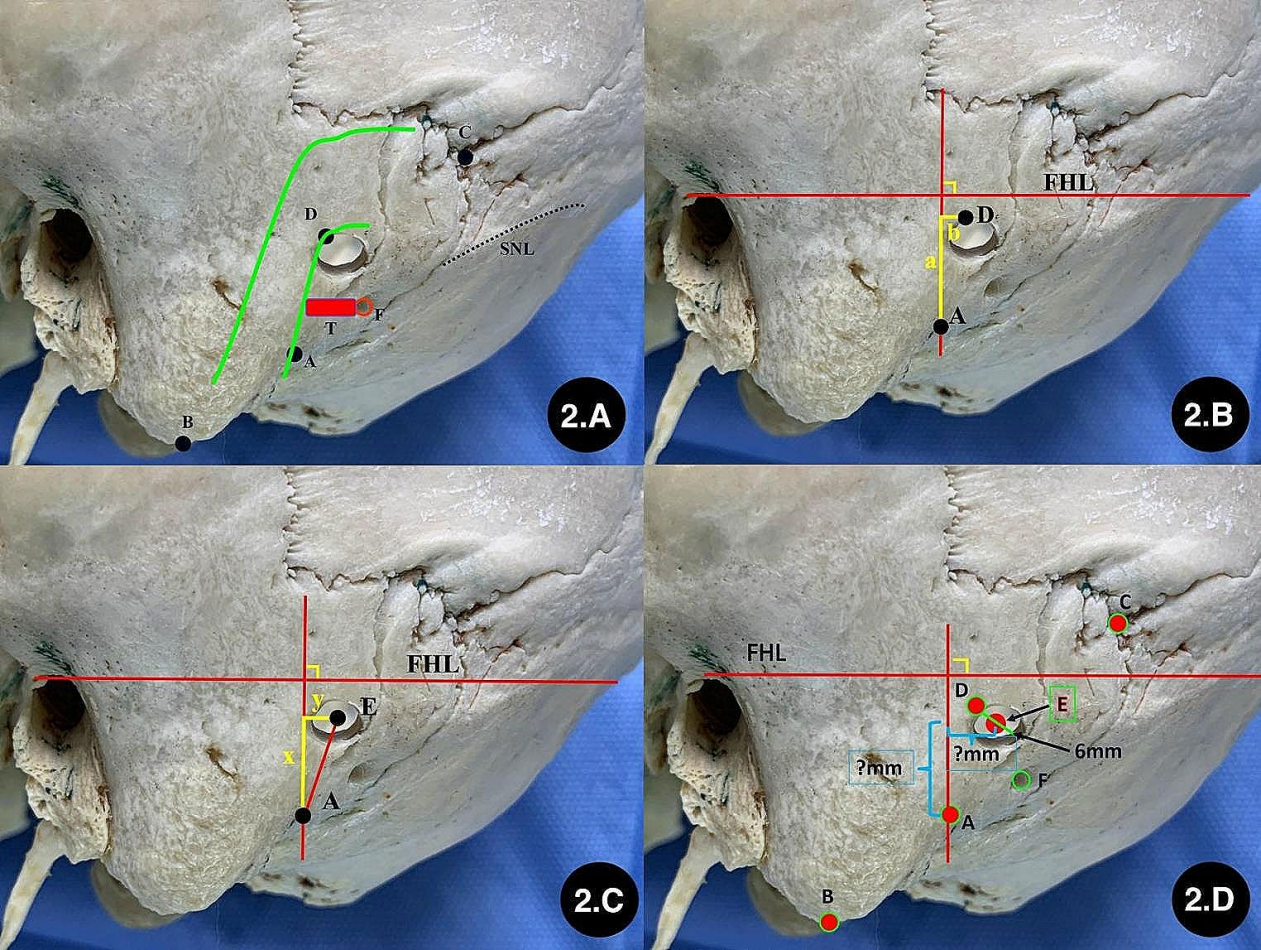
Establishing the coordinate system for locating “keypoint” and “keyhole”. **A**: observing the relationship between burr hole and TSJ from skull surface; **B**: measuring the position between point A and D in the coordinate system; **C**: measuring the position between point A and E in the coordinate system; **D**: concept of locating the central point of “keyhole” in the coordinate system. A: the top point of the digastric groove; B: mastoidale; C: asterion; D: keypoint; E: the central point of “keyhole”; F: mastoid foramen; ;SNL: superior nuchal line; FHL: the baseline between the infraorbital margin and superior margin of the external acoustic meatus; T: tube of emissary vein; a: the perpendicular distance between D and A in the coordinate system; b: the horizontal distance between D and A in the coordinate system; x: the perpendicular distance between E and A in the coordinate system; y: the horizontal distance between E and A in the coordinate system

1. Location of the inferior margin of the keypoint: The inferior margin of the transverse sinus sulcus and the posterior margin of the sigmoid sinus sulcus were identified on the internal surface of the skull.The tangents of the inferior margin of the transverse sinus sulcus and the posterior margin of the sigmoid sinus sulcus were marked and intersected at point K. The point at which the bisector of the inferoposterior angle of that pair intersects with the inferoposterior margin of the sinus sulcus was defined as the keypoint (Fig. 1 and A).
2. Drilling the “keyhole”: Taking the TSJ as the reference point, a bone hole (diameter: 6 mm) was drilled perpendicular to the skull from inside to outside, and this hole was defined as the “keyhole,” which is the burr hole in craniotomy (Fig. 1 and B).
3. The line between the infraorbital margin and superior margin of the external acoustic meatus (ISL) was defined as the baseline, which was the extracranial projection line of the Frankfort horizontal plane (FHL) (Fig. 1 and C).
4. Marking extracranial anatomical landmarks: The top point of the digastric groove (A) and the mastoidale (B), asterion (C), keypoint (D), central point of the “keyhole” (E), mastoid foramen, (F) and superior nuchal line (SNL) were marked on the external surface of the skull (Fig. 1 and D).
5. The lengths of AD, BD, and CD were measured (Fig. 1 and E).
6. The anteroinferior angle (∠α) between AD and baseline was measured (Fig. 1 and F).
7. To observe the relationship between anatomical marker and junction of transevers sinus and sigmoid sinus from surface. (Figure 2 and A);
8. A coordinate system was established using the baseline and its perpendicular line through point A. The perpendicular distance (a) and horizontal distance (b) between the keypoint and the top point of the digastric groove in the coordinate system were measured (Fig. 2 and B).
9. The length of AE, the perpendicular distance (x), and the horizontal distance (y) between the central point of the “keyhole” and the top point of the digastric groove in that coordinate system were measured (Fig. 2, C and D).

#### Imitation and evaluation of the retrosigmoid “keyhole” approach on cadaveric specimens to verify the observations in the dry skulls

**Fig. 3:**
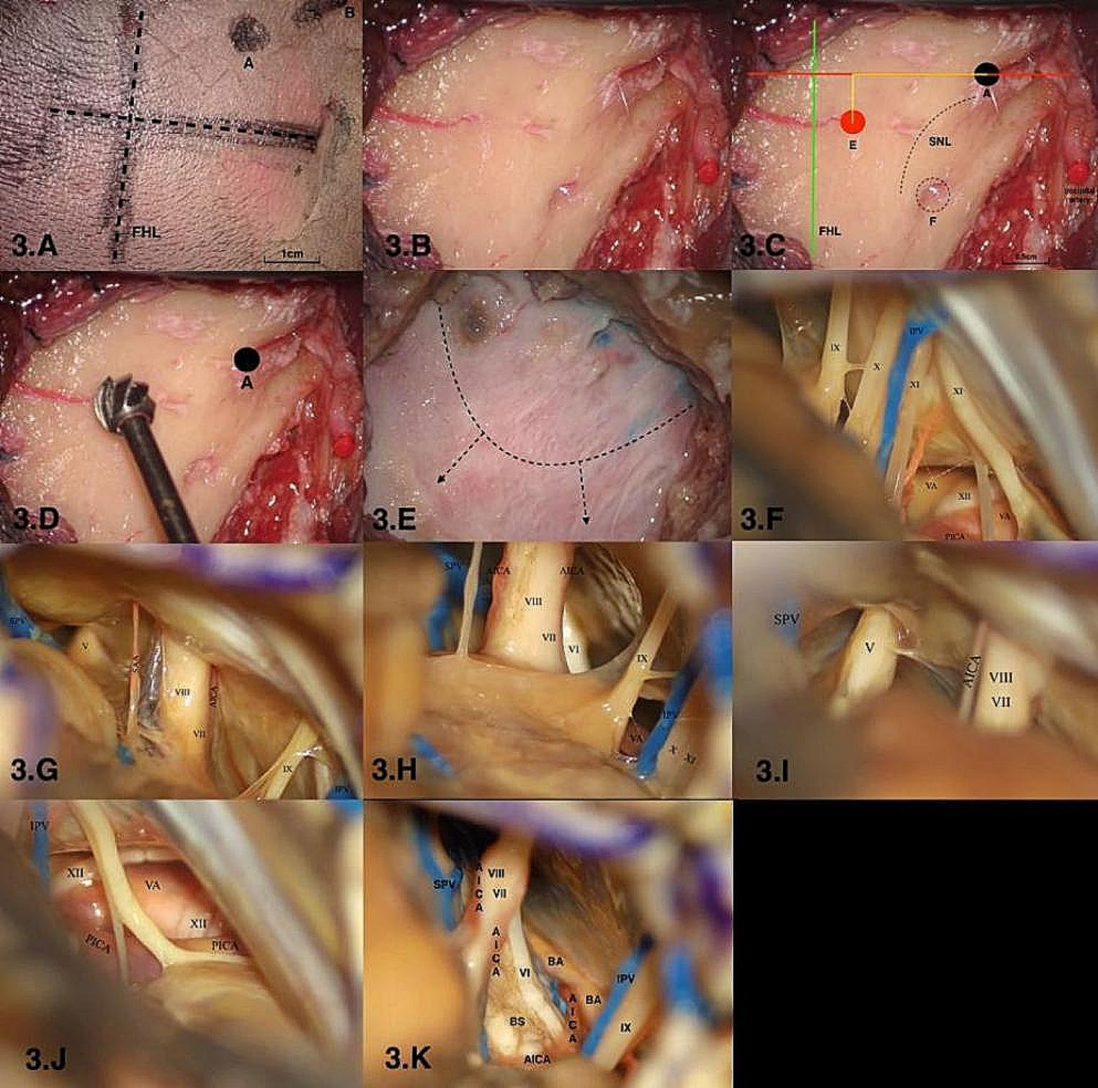
Imitating and evaluating the new craniotomic method of the retrosigmoid keyhole approach on cadaveric specimens. **A**: Incision of the scalp; **B**: recognize the bone landmarks; **C**: establishing the coordinate system and locate the drilling point; **D**: drill a burr hole; **E**: incision of dura; **F**-**K**: Observe the cranial nerve and vessels through the suboccipital retrosigmoid keyhole approach via “one point, two lines and two distances” theory; A: the top point of the digastric groove; E: the central point of “keyhole”; F: mastoid foramen; G: occipital artery; FHL: the baseline between the infraorbital margin and superior margin of the external acoustic meatus; SNL: superior nuchae line; SPV: superior petrosal vein; IPV: inferior petrosal vein; V: trigeminal nerve; VI: abducent nerve; VII: facial nerve; VIII: acoustic nerve; CNIX: Glossopharyngeal nerve; X: vagus nerve; XI: accessory nerve; XII: hypoglossal nerve; AICA: anterior inferior cerebellum artery; PICA: posterior inferior cerebellum artery; BA: basal artery; VA: vertebral artery; BS: brain stem

A line was drawn between the infraorbital margin and superior margin of the external acoustic meatus to represent the baseline. A straight incision measuring approximately 4 cm was made perpendicular to the baseline with 1.25 cm posterior to the top point of the digastric groove. Approximately one-quarter of the incision was made above the baseline. The inferior border of the incision reached the level of the mastoidale (Fig. 3 and A). 2. The top point of the digastric groove and mastoid foramen were identified on the surface of the skull (Fig. 3 and B). 3. A coordinate system was established using the baseline and its perpendicular line through point A (Fig. 3 and C). 4. The drilling point was confirmed according to previous observations in the dry skulls: 14.0 mm above the top point of the digastric groove and 6.5 mm behind the top point of the digastric groove in the coordinate system (Fig. 3 and D). 5. Cut-up the dura in an arc shape (Fig. 3 and E). (6) The operative area was exposed to evaluate whether the exposed area was sufficient (Fig. 3, F and K).(video 1.anatomical video).

#### Clinical application of accurate craniotomy in the retrosigmoid approach, based on an anatomical study

**Fig. 4:**
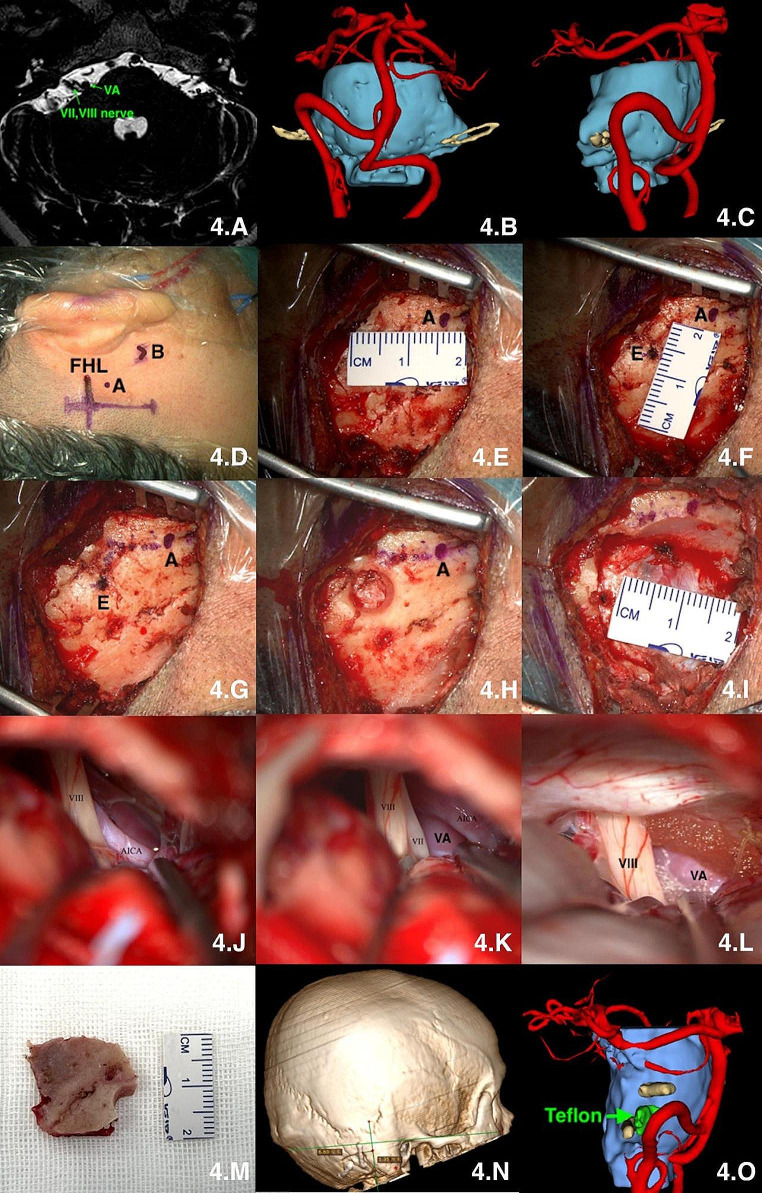
microsurgical treatment of Hemifacial spasm via suboccipital retrosigmoid keyhole approach based on “one point, two lines and two distances” theory. **A**: MR scan; **B** and **C**: multimodal reconstruction of VII nerve and AICA; **D**: Incision; **E**: confirming the top point of the digastric groove“A” and measuring the “x” distance; **F**: measuring the “y” distance; **G**: confirming the central point of keyhole “E”; **H**: drilling burr hole; **I**: size of bone window; **J**: observing the relationship between VII nerve and AICA; **K**: observing the relationship between VII nerve and VA; **L**: Detach with teflon; **M**: size of bone flap; **N** and **O**: postoperative 3D reconstruction. A: the top point of the digastric groove; B: mastoidale; FHL: the baseline between the infraorbital margin and superior margin of the external acoustic meatus; E: the central point of “keyhole”; VII: facial nerve; VIII: acoustic nerve; AICA: anterior inferior cerebellum artery; VA: vertebral artery

**Fig. 5:**
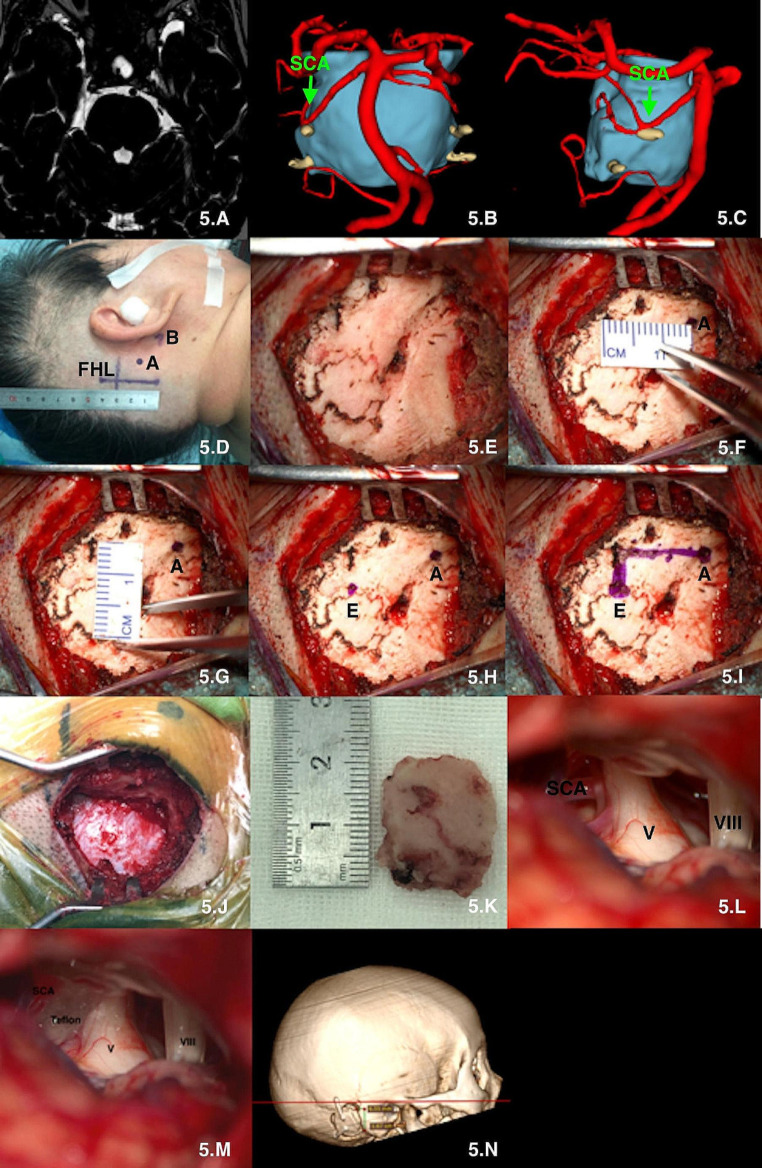
microsurgical treatment of Trigminal neuralgia via suboccipital retrosigmoid keyhole approach based on “one point, two lines and two distances” theory. **A**: MR scan; **B** and **C**: multimodal reconstruction of V nerve and SCA; **D**: Incision; **E**: exposure of bone surface; **F**: confirming the top point of the digastric groove “A” and measuring the “x” distance; **G**: measuring the “y” distance; **H**-**I**: confirming the central point of keyhole “E”; **J**: size of bone window; **K**: size of bone flap; **L**: observing the relationship between V and SCA; **M**: Distract with teflon; **N**: postoperative 3D reconstruction. A: the top point of the digastric groove; E: the central point of “keyhole”; V: trigeminal nerve; VIII: acoustic nerve; SCA: superior cerebellum artery

**Fig. 6:**
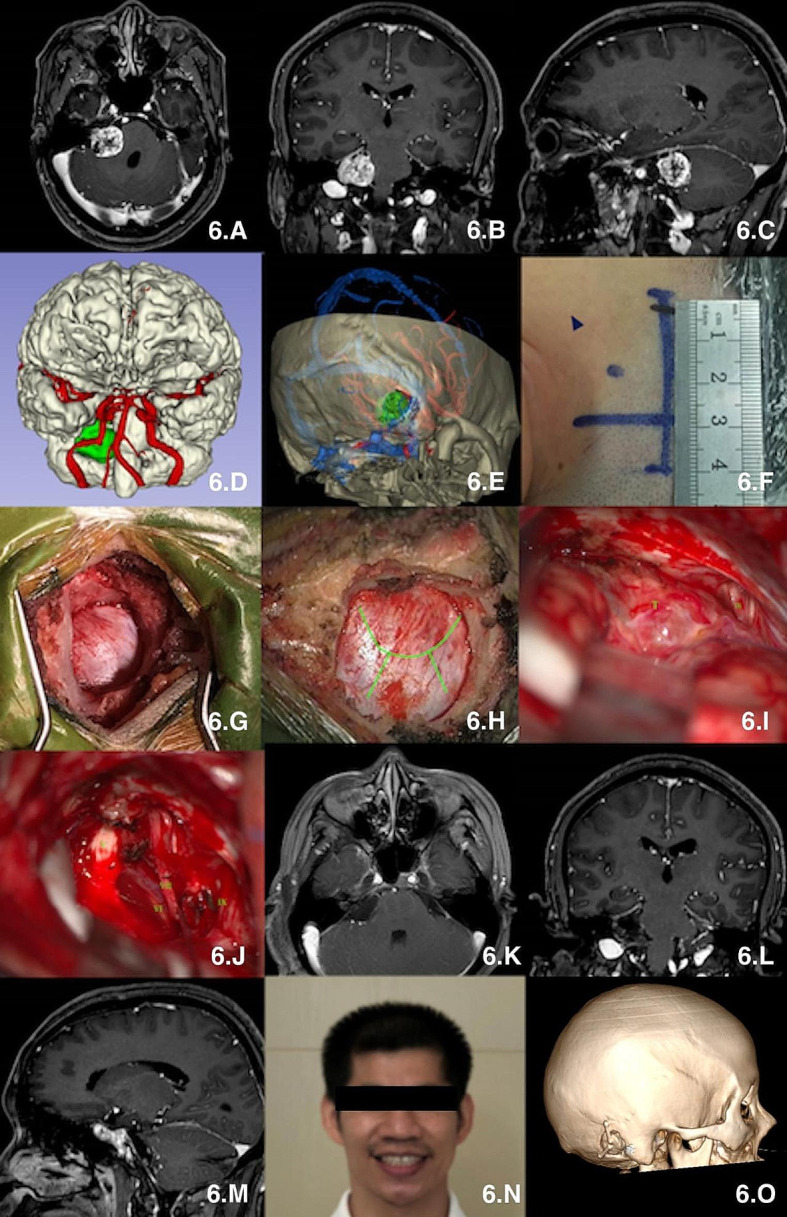
microsurgical treatment of vestibular neuroma via suboccipital retrosigmoid keyhole approach based on “one point, two lines and two distances” theory. **A**-**C**: MR scan; **D** : multimodal reconstruction of tumor, vessels and brain tissue; **E**: simulation approach using multimodal reconstruction; **F**: Incision; **G**: exposure of bone window; **H**: incision of dura; **I**:exposure of tumor; **J**: observation after tumor resection; **K**-**M**: postoperative MR scan; **N**: patient with no neurological deficit after surgery; **O**:postoperative 3D reconstruction of skull. V: trigeminal nerve; VI: abducent nerve; VII: facial nerve; IX: Glossopharyngeal nerve; T:tumor

(1) All the operations were performed under general anesthesia with endotracheal intubation and completed by one experienced neurosurgeon. Multimodal image fusion and 3D reconstruction were performed to simulate the surgical procedure. (Figures 4, B and C, 5, B and C and 6, D and E).(2) The baseline (the line between the infraorbital margin and superior margin of the external acoustic meatus) was drawn and the mastoid tip and the top point of the digastric groove were marked on the skin surface. A straight incision of approximately 4–5 cm in length was made perpendicular to the baseline with 1.25 cm posterior to the top point of the digastric groove.(Figures 4 and D, 5 and D and 6 and F). (3) The scalp and muscles were cut open to completely expose the digastric groove, emissary vein, and other structures (Figs. 4 and G, 5 and E and 6 and G).(4) A coordinate system was established using the baseline and its perpendicular line through the top point of digastric groove. The centre point of “burr hole” was confirmed according to our theory: 14.0 mm above the top point of the digastric groove and 6.5 mm behind the top point of the digastric groove in the coordinate system. Burr hole were created by a drill, and the bone flap was dissected using a milling cutter to reveal the posterior margin of the sigmoid sinus and the distal inferior margin of the transverse sinus (Figs. 4 and I, 5 and J and 6 and H). (5) The dura was opened microscopically to fully expose the surgical area and proceed to the next surgical procedure. (Figures 4, J, K and L, 5, L and M and 6, I and J). (6) The dura was closed tightly after the craniotomies were completed, and the bone flap was fixed with connecting sheet, and the muscle and scalp were sutured. Brain CT and MR images were reviewed postoperatively (Figs. 4, N and O, 5 and N and 6 and O). (video 2.clinical application)

### Statistical analysis

The distances and angles between related structures were measured using a digital Vernier caliper and a digital display angle scale. The results were expressed as mean ± standard deviation (SD). Statistical analyses were performed using Student’s t-tests (SPSS for Windows, Version 21.0; IBM Corporation, Armonk, NY, USA), with significance set at p-value < 0.05.

## Results

The distances between the keypoint (D) and the relevant anatomical landmarks are listed in Table 1. The values of AD, BD, and CD were 17.42 ± 2.15 mm, 36.33 ± 2.90 mm, and 15.99 ± 3.99 mm, respectively, on the left and 18.13 ± 2.13 mm, 36.75 ± 2.66 mm, and 16.34 ± 3.96 mm, respectively, on the right (*P* > 0.05). The value of ∠α was 73.49 ± 8.22° on the left and 73.93 ± 7.16° on the right (*P* > 0.05). The values of a and b were 16.60 ± 2.53 mm and 4.75 ± 1.99 mm, respectively, on the left and 17.34 ± 2.46 mm and 4.85 ± 1.75 mm, respectively, on the right (*P* > 0.05)(Table 2). The value of AE was 15.82 ± 2.02 mm on the left and 16.51 ± 2.01 mm on the right (*P* > 0.05). The values of x and y were 14.20 ± 2.63 mm and 6.54 ± 1.83 mm, respectively, on the left and 14.95 ± 2.53 mm and 6.65 ± 1.61 mm, respectively, on the right, (*P* > 0.05) (Table 3).

**Table 2:** Relationship between the keypoint and relevant anatomical markers (mm, °)

	AD	BD	CD	∠α	a	b
LT	17.42 ± 2.15	36.33 ± 2.90	15.99 ± 3.99	73.49 ± 8.22	16.60 ± 2.53	4.75 ± 1.99
RT	18.13 ± 2.13	36.75 ± 2.66	16.34 ± 3.96	73.93 ± 7.16	17.34 ± 2.46	4.85 ± 1.75
t	-0.937	-0.428	-0.254	-0.161	-0.847	-0.154
P	0.356	0.672	0.801	0.874	0.404	0.878

AD: distance between the keypoint and the top point of the digastric groove; BD, distance between the keypoint and the mastoidale; CD, distance between the keypoint and the asterion; ∠α: anterior-inferior angle between AD and the baseline; a: perpendicular distance between D and A in the coordinate system; b: horizontal distance between D and A in the coordinate system

**Table 3:** Relationship between the borehole and relevant anatomical markers (mm)

	AE	x	y
LT	15.82 ± 2.02	14.20 ± 2.63	6.54 ± 1.83
RT	16.51 ± 2.01	14.95 ± 2.53	6.65 ± 1.61
t	-0.968	-0.825	-0.185
P	0.341	0.341	0.855

AE: straight distance from the top point of the digastric groove to the center of the burr hole; x: perpendicular distance between E and A in the coordinate system; y: horizontal distance between E and A in the coordinate system

In all five fresh cadaveric specimens, the craniotomy method using skull specimens had the following results: (1) Accuracy: Satisfactory exposure of important structures; (2) Safety: Damage to venous sinuses due to drilling was not observed; (3) Minimal invasiveness: The mean diameter of the bone windows ranged from 1.8 to 2.5 cm and 2.1 to 3.0 cm.

Clinical application: In 53 patients who underwent craniotomy, we verified the accuracy, safety, minimal invasiveness, and rapidity of our methods. (1) Accuracy: The transverse and sigmoid sinuses of 47 patients were well-exposed. The sinuses of the other six patients were also well-exposed after adequate bone grinding. The local microanatomic structures of the operative fields were well-exposed after the dura was cut. (2) Safety: No drilling-related injury to the venous sinuses was observed. The sigmoid sinus of one patient was injured because of poor handling of emissary veins during the formation of the bone flap. The injury was controlled and minimized immediately and had no relationship with the location method. (3) Rapidity: The mean craniotomy time was 26.01 ± 3.46 min. (4) minimal invasiveness: The mean diameter of the bone windows ranged from 2.0 to 2.5 cm. The bone flap was restored (Table 4).

**Table 4:** Clinical data of the 53 patients

Patient	Sex	Age	Diagnosis	Bone window (mm)	Craniectomy time (min)	Sinus damage	Surgical view adequacy
***	M	32	HFS	25*25	22.12	N	Y
***	F	33	HFS	20*30	26.38	N	Y
***	M	35	HFS	20*20	33.23	N	Y
***	F	39	HFS	20*30	22.32	N	Y
***	M	40	HFS	25*40	23.58	N	Y
***	F	41	HFS	20*25	26.38	N	Y
***	M	42	HFS	25*25	22.37	N	Y
***	F	43	HFS	20*31	25.26	N	Y
***	F	45	HFS	20*20	23.54	N	Y
***	M	45	HFS	20*30	28.48	N	Y
***	F	50	HFS	20*30	27.54	N	Y
***	M	51	HFS	30*35	24.52	N	Y
***	M	52	HFS	25*25	25.22	N	Y
***	M	53	HFS	25*25	23.23	N	Y
***	F	53	HFS	20*30	24.56	N	Y
***	M	54	HFS	20*20	31.22	N	Y
***	M	54	HFS	25*25	35.41	N	Y
***	M	55	HFS	20*20	24.33	N	Y
***	M	58	HFS	25*20	30.12	N	Y
***	M	58	HFS	20*20	26.58	N	Y
***	F	59	HFS	20*20	22.35	N	Y
***	F	60	HFS	25*25	27.43	N	Y
***	F	62	HFS	25*20	33.52	N	Y
***	F	65	HFS	25*20	25.34	N	Y
***	F	69	HFS	20*20	29.12	N	Y
***	M	39	TN	25*25	21.32	N	Y
***	F	40	TN	25*25	25.33	N	Y
***	F	41	TN	30*25	21.12	N	Y
***	F	43	TN	30*25	31.53	N	Y
***	M	43	TN	30*25	25.23	N	Y
***	M	45	TN	20*30	27.43	N	Y
***	F	46	TN	30*25	27.43	N	Y
***	F	49	TN	20*30	33.42	N	Y
***	M	50	TN	25*20	25.23	N	Y
***	F	53	TN	20*30	22.53	N	Y
***	F	53	TN	30*25	23.38	N	Y
***	F	57	TN	30*25	29.32	N	Y
***	F	58	TN	30*30	21.32	N	Y
***	M	62	TN	30*30	22.47	N	Y
***	M	64	TN	25*25	22.47	N	Y
***	F	68	TN	20*20	27.59	N	Y
***	F	68	TN	30*30	23.22	N	Y
***	F	68	TN	30*30	28.54	N	Y
***	F	72	TN	20*30	31.22	N	Y
***	F	29	Acoustic neuroma	20*31	22.57	N	Y
***	F	36	Acoustic neuroma	20*30	22.35	N	Y
***	F	36	Acoustic neuroma	30*25	27.54	N	Y
***	M	37	Acoustic neuroma	25*25	24.56	N	Y
***	F	51	Acoustic neuroma	25*40	24.47	N	Y
***	M	53	Acoustic neuroma	20*30	25.26	N	Y
***	F	63	Acoustic neuroma	30*25	23.58	N	Y
***	F	37	Schwannoma	20*30	27,57	N	Y
***	F	48	Meningioma	30*25	24.52	N	Y

TN: trigminal neuralgia; HFS: hemifacial spasm

## Discussion

The suboccipital retrosigmoid approach is used to treat cerebellopontine angle lesions [9–12]. With the concept of minimally invasive neurosurgery [13, 14], the asterion has been considered an important landmark for locating keypoints [15–17]. However, an increasing number of studies have shown that anatomical variations in the asterion are common. Some reports have suggested that there is anatomic variation in the asterion: around 85% asterions were exactly at the lateral side of the transverse sinus, while about 10% asterions were above the transverse sinus and only 5% were below the transverse sinus [18–20]. The asterion is often posterior superior to the key point. Therefore, some scholars cautioned against the risks of injuring the venous sinus when using the asterion as a key point in craniotomy. At the same time, owing to the limitation of “keyhole” incision which is only 4 cm, the asterion cannot be properly exposed, making it unsuitable for locating the keypoint. Therefore, accurate identification of the keypoint on the external surface of the skull, with limited anatomical landmarks in the operative area, has become a research focus.

The development of imaging techniques has provided neurosurgery with many new and accurate positioning methods, and can also advance knowledge of individual variability to formulate personalized surgical approaches, reducing surgery-related injuries and complications [21–23]. However, these methods require certain equipment and facilities, high personnel requirements, complex preoperative planning processes, and increased financial burden on patients, which have not allowed their widespread clinical application. Therefore, there is a need to find a simple, accurate, and reliable method to locate the keyhole and guide precise craniotomy through the retrosigmoid keyhole approach.

**Fig. 7:**
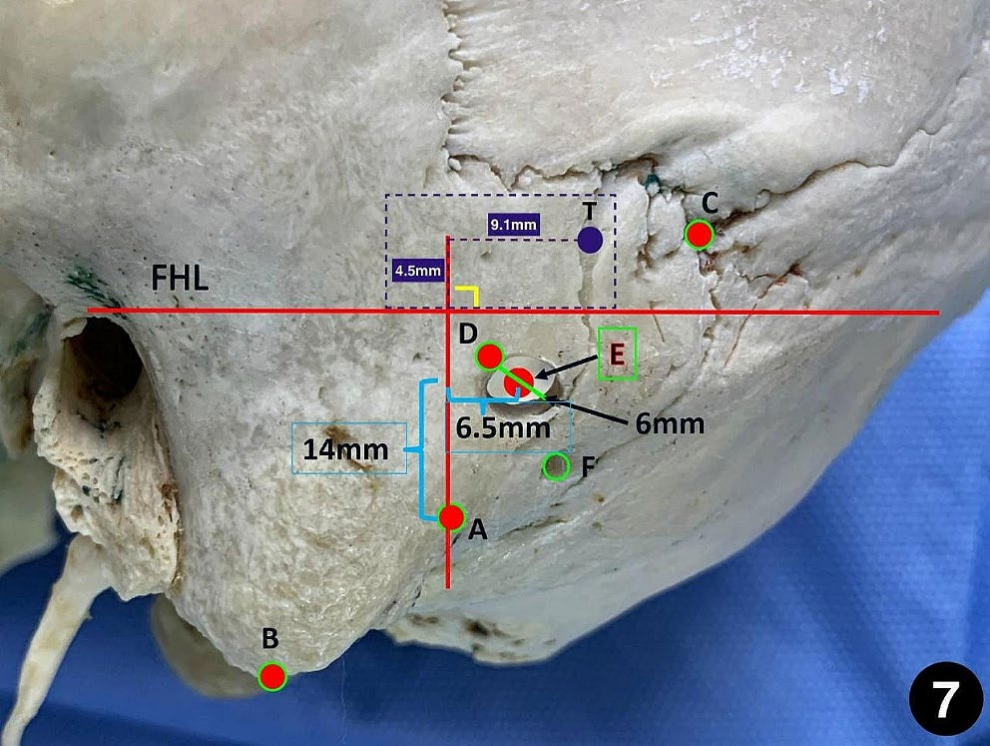
the method of precisely locating the central point of keyhole via suboccipital retrosigmoid keyhole approach based on “one point, two lines and two distances” theory. A: the top point of the digastric groove; B: mastoidale; C: asterion; D: keypoint; E: the central point of “keyhole”; F: mastoid foramen; FHL: the baseline between the infraorbital margin and superior margin of the external acoustic meatus; T: burr hole identification base on Tubbs’ theory

Tubbs have studied various skull surface markers and their relationships and he established a coordinate system to evaluate these landmarks. The distances of burr hole and the “zygomatic line”, “mastoid line” were calculated. However, when we use Tubb’s theory for burr hole localization in some clinical cases, we found that burr holes were usually above or on the surface of the transverse sinus. therefore, a risk of venous sinus damage was identified. Inspired by Tubbs’ study, we found that the top point of the digastric groove is an important and reliable bony marker. It is located in the surgical area of a minimally invasive incision and has a close relationship with the transverse sinus and sigmoid sinus [24–26]. On the other hand, the top point of the digastric groove is easy to find intraoperatively, so we have chosen it as an anatomical landmark to locate keypoint. In order to accurately measure the relationship between the top point of the digastric groove and the keypoint, we took the classical Frankfurt horizontal plane projection (FHL) on the sagittal plane as the X-axis, and the projection of the top point of the digastric groove as the vertical line to form the Y-axis, and established the coordinate system. Meanwhile, we also projected the keypoint vertically into the coordinate system, so as to calculate the relationship between the keypoint and the top point of the digastric groove. That is, the key point is above (16.60 ± 2.53)mm and behind (4.75 ± 1.99)mm(left), above (17.34 ± 2.46)mm and behind (4.85 ± 1.75)mm(right) of the top point of digastric groove. However, in clinical applications, surgeons are more concerned about the location of the center point of key hole. We can determine that the center point of the keyhole is on the circle inferior and medial with a radius of 3 mm with the keypoint as the center of the circle. Therefore, by using the same coordinate system, we can directly measure and locate the position of the center point of the keyhole. That is, the “center point of keyhole” is located above (14.20 ± 2.63)mm and medial (6.54 ± 1.83)mm(left), above (14.95 ± 2.53)mm and medial (6.65 ± 1.61)mm(right) of the top point of digastric groove. If the burr hole with a diameter of about 6 mm is drilled at this point, it can be closest to the key point and minimize the probability of damage to the transverse sinus and sigmoid sinus. Then, a bone window with a diameter of about 20 ~ 25 mm is made by using a milling cutter through the top point of the digastric groove, which can fully meet the requirements of exposure of intracranial anatomical structure. We define this new keyhole positioning theory as “one point, two lines, two distances theory”. Using the baseline and the coordinate system, which is established with the baseline (FHL, the line between the infraorbital margin and superior margin of the external acoustic meatus) and its perpendicular line through the top point of the digastric groove. The central point of the “keyhole” is 14.0 mm above and 6.5 mm behind the top point of the digastric groove in the coordinate system. The keypoint is located approximately 3–4 mm medial-superior to the central point of the “keyhole.” (Fig. 7).

We selected 5 fresh cadaver heads to verify the above positioning and craniotomy methods. Based on the results of simulated craniotomy surgery, the accuracy, safety, speed, and minimally invasive nature of the surgery all achieved satisfactory results.

Finally, we applied our original positioning and craniotomy methods to 53 clinical cases for surgical treatment, and no cases of venous sinus damage were reported during the operation. In 47 cases, the drilling point was accurately located, and in the remaining 6 cases, satisfactory exposure was obtained after mild grinding. Referring to a previous study, the mean operative time in the common approach from skin to posterior fossa was 30 min (15 to 42 min) [27]_,_ Our study (26.01 ± 3.46 min) showed a shorter craniectomy time.

Locating the keypoint assisted by emissary vein and SNL is feasible. Anatomic variations are observed in the mastoid foramen. The mean diameter of the mastoid emissary vein foramen is 2.15 ± 0.8 mm occurring at a rate of 61%. The significantly high rate of variability of the mastoid foramen leads to difficulty in precisely locating the keypoint. However, the keypoint is always anterosuperior to the mastoid foramen. Additionally, the inner opening of the mastoid emissary vein is located at the vertical part of the sigmoid sinus [28]. The lateral part of the SNL is located below the level of the transverse sinus and the length between the SNL and transverse sinus ranges from 1.5 mm to 14 mm [29]. These modifications provide another reference to locate the keypoint: anterosuperior to the mastoid foramen and above the lateral portion of the SNL.

Disadvantages and shortcomings: (1) Failure to classify skull specimens according to sex may have affected the results of the present study. (2) Second, there would be variations when this method is applied in clinical settings. Therefore, more application cases and preoperative multi-modal image fusion technology are required to develop the method.

## Conclusions

This localization method we named “one point, two lines, and two distances” theory. “one point” is the top point of the digastric groove. “two line” is the baseline between the infraorbital margin and superior margin of the external acoustic meatus and the perpendicular line to the baseline through the top point of the digastric groove. “two distances” is the perpendicular and horizontal distances between the centre point of keyhole and the top point of the digastric groove in the coordinate system. The spatial positions of the keypoint and drilling point can be confirmed using the coordinate system. The drilling point was 14.0 mm above and 6.5 mm behind the top point of the digastric groove in the coordinate system. The keypoint was located approximately 3–4 mm medial-superior to the drilling point. Our localization method is simple, safe, operable, and practical. Further, this method can provide a quantitative anatomical basis for safe, accurate, and rapid craniotomy.

## Electronic supplementary material

Below is the link to the electronic supplementary material.

**Supplementary Material 1: Video 1** Imitation the retrosigmoid keyhole approach on cadaveric specimens viaone point, two lines, two distances theory

**Supplementary Material 2: Video 2** Clinical application of accurate craniotomy in the retrosigmoid keyhole approach, based on one point, two lines, two distances theory

## Data Availability

All the experimental data and the simulation results that support the findings of this study are original and available in BaiduNetdisk (https://pan.baidu.com/s/1wjBurVZFKgB37dOzCSh25w).
